# An electronic health record (EHR) log analysis shows limited clinician engagement with unsolicited genetic test results

**DOI:** 10.1093/jamiaopen/ooab014

**Published:** 2021-03-01

**Authors:** Jordan G Nestor, Alexander Fedotov, David Fasel, Maddalena Marasa, Hila Milo-Rasouly, Julia Wynn, Wendy K Chung, Ali Gharavi, George Hripcsak, Suzanne Bakken, Soumitra Sengupta, Chunhua Weng

**Affiliations:** 1 Department of Medicine, Division of Nephrology, Columbia University, New York, New York, USA; 2 The Irving Institute for Clinical and Translational Research, Columbia University, New York, New York, USA; 3 Department of Medicine, Center for Precision Medicine and Genomics, Columbia University, New York, New York, USA; 4 Department of Pediatrics, Columbia University, New York, New York, USA; 5 Departments of Pediatric and Medicine, Columbia University, New York, New York, USA; 6 Department of Biomedical Informatics, Columbia University, New York, New York, USA

**Keywords:** clinical engagement with genomic results, electronic health records, log analysis

## Abstract

How clinicians utilize medically actionable genomic information, displayed in the electronic health record (EHR), in medical decision-making remains unknown. Participating sites of the Electronic Medical Records and Genomics (eMERGE) Network have invested resources into EHR integration efforts to enable the display of genetic testing data across heterogeneous EHR systems. To assess clinicians’ engagement with unsolicited EHR-integrated genetic test results of eMERGE participants within a large tertiary care academic medical center, we analyzed automatically generated EHR access log data. We found that clinicians viewed only 1% of all the eMERGE genetic test results integrated in the EHR. Using a cluster analysis, we also identified different user traits associated with varying degrees of engagement with the EHR-integrated genomic data. These data contribute important empirical knowledge about clinicians limited and brief engagements with unsolicited EHR-integrated genetic test results of eMERGE participants. Appreciation for user-specific roles provide additional context for why certain users were more or less engaged with the unsolicited results. This study highlights opportunities to use EHR log data as a performance metric to more precisely inform ongoing EHR-integration efforts and decisions about the allocation of informatics resources in genomic research.

**LAY SUMMARY**

To evaluate whether clinicians at our institution viewed eMERGE study participants genetic test results entered in the EHR, we analyzed users’ EHR access logs. We found clinician users viewed only 1% of all the EHR-integrated eMERGE results. We also identified several patterns of user engagement with the EHR’s *Genetics* section and with eMERGE genetic test results based on the user’s clinical role, practice setting, and the context for their involvement in a participant’s care.

## INTRODUCTION

The success of Precision Medicine initiatives relies, in large part, on understanding how clinicians utilize genomic data. Several NIH-funded initiatives, such as the Clinical Sequencing Evidence-generating Research (CSER) consortium,^1^ Implementing GeNomics in practice (IGNITE),^2^ and the Electronic Medical Records and Genomics (eMERGE) Networks,^3^ have set out to assess how clinicians engage with medically actionable genomic findings. Within these initiatives, members of the eMERGE Network—a NHGRI-funded consortium of academic and integrated health systems established in 2007 (https://emerge-network.org)—have invested institutional resources into electronic health record integration. To date, these efforts have primarily focused on the electronic transmittal of standardized genomic data from laboratories to healthcare providers.^4^ Despite dedicated efforts across these sites to display actionable genomic data in the EHR, it remains to be seen *if* clinicians utilize unsolicited genomic findings in their medical decision-making, and whether it impacts patient care. EHR access and audit logs offer unique opportunities to scale observational research on user behaviors in a disease-agnostic and minimally biased manner.^5–7^ To date, no attempts have been made to assess clinician engagement with genomic data using EHR access logs.^8–14^ Therefore, we set out to evaluate the breadth of clinicians’ engagement with unsolicited EHR-integrated genetic test results of eMERGE participants using automatically generated access logs within a large tertiary care academic medical center.

## MATERIALS AND METHODS

The eMERGE^3^ Network’s phase III study set out to return medically actionable genomic findings to 25 380 biobank participants and integrate the data into the EHR to support personalized medicine efforts and to track long-term outcomes. Participants underwent clinical-grade genetic screening with a customized next-generation sequencing panel (ie, eMERGE-*Seq* platform) of 109 clinically relevant, and medically actionable genes^15^^,^^16^. Between 2016 and 2017, 1071 eMERGE participants were prospectively enrolled at New York-Presbyterian Hospital (NYP)/Columbia University Irving Medical Center.^17^^,^^18^ The study’s return of results phase began October 1, 2018–May 31, 2019. In total, 1021 participants with “negative” (ie, nondiagnostic) results were mailed a genetic counseling letter and a copy of the molecular pathology report; 50 participants with “positive” (ie, genetic variants classified as Pathogenic or Likely Pathogenic, per the American College of Medical Genetics and Genomics,^19^ or a genetic risk variant deemed actionable) results were invited to return for an in-person genetic counseling. Genetic test results and counseling letters were entered (*.pdf*) in the *Genetics* section of a home-grown EHR (iNYP).^20^

This study did not include human subjects. This purely observational (ie, noninterventional) research involved the analysis of materials (eg, data, documents, records or specimens) collected solely for nonresearch purposes (eg, medical treatment, diagnosis, etc.). The risk of harm to subjects exposed as a result of this research was no more than minimal.

### Data sources and preprocessing

iNYP, a hospital-supported web-based platform, displays patients aggregated clinical information, and is accessed directly through its website and through both the hospital- (*SCM Allscripts*) and university-supported (*Crown*) EHR systems. iNYP was the platform selected to input eMERGE results because of its flexibility for developing test result display functions, popularity among users and its easy-to-use reputation.^21^^,^^22^

In the context of log analysis, we defined “clinician engagement” with genetic test results as any episode of patient record access by a clinician that involved an interaction with the *Genetics* section (and any document within this section) of the iNYP platform—for example, an attempt to access the *Genetics* section, an attempt to view the genetic test report, etc. A logged user session was defined as a recorded set of unique user’s interactions with iNYP that involved the *Genetics* section within a single platform login/logout (or timeout due to inactivity) period. The degree of clinician's engagement with results was assessed based on the number and type of documents (ie, the pathology report and/or counseling letter) accessed in the *Genetics* section, along with the amount of time spent on this activity (also, see *Data analysis*).

**Figure 1.**
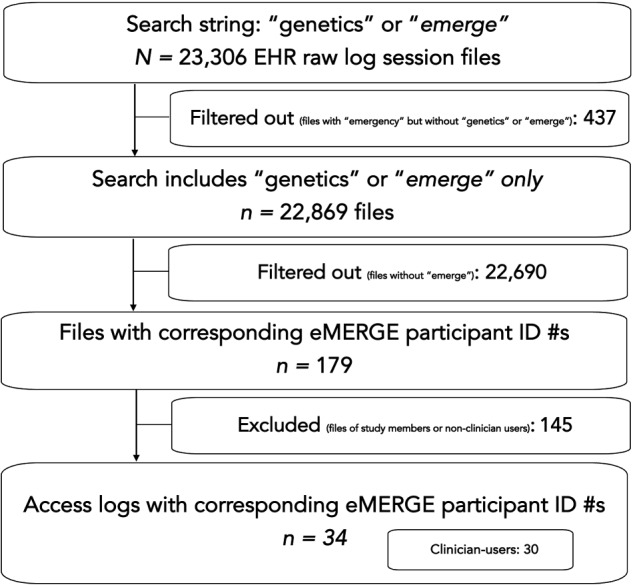
Study flowchart.

A custom-programmed script was used to retrieve iNYP raw log session files of all hospital- and university-affiliated users who viewed any type of genetic data in iNYP from October 1st, 2018 to December 16th, 2019. Logs captured user access of iNYP’s *Genetics* section, including users who accessed it through the university- and hospital-supported EHRs. The following elements were captured in each log file: relevant search terms (eg, “genetics,” “emerge,” etc.), date and time of access, user login, EHR platform used to access iNYP, patient medical record number, and when applicable—a unique eMERGE identification number and a timestamp of the genetic test result document viewed. Initially, 23 306 raw logs session files were retrieved (Figure 1) using this script. We then removed 437 files that contained “emergency.” The 22 869 files were of all users who accessed iNYP’s *Genetics* section. Next, we filtered out 22 690 files that did *not* contain “emerge” and the corresponding eMERGE identification number. The remaining 179 files were of users who accessed the *Genetics* section of an eMERGE participant. Then, 145 files were excluded as the users were study team members or nonclinicians (eg, IT employees, third-party contractors, administrators, etc.). Adjudication of nonclinician users was based on their university/hospital-designated employee title and absence of a *National Provider Identification* number. For the remaining access logs, we manually identified each user and categorized each of them by gender, employer (eg, hospital or university), clinical role (ie, attending/fellow/resident-level physicians vs. nurse practitioners), specialty (ie, medicine vs. nonmedicine disciplines) using employee directories. Then, a clinician (JGN) performed in-depth chart reviews to determine the context in which each user accessed the EHR’s *Genetics* section of an eMERGE participant.

### Data analysis

Clustering analysis, long recognized as an effective classification method for exploratory analysis of high-dimensional and heterogenous EHR log data,^23^^,^^24^ was used to identify patterns of clinicians’ engagement with EHR-integrated genetic results. Variables extracted from access logs and participants’ charts included in the final dataset used for the clustering analysis were as follows: time of *Genetics* section access, EHR platform used to access iNYP, practice setting, clinician categorical data, type of genetic test result document opened and clinical context of *Genetics* section access. Given the mixed-type data, along with the lack of *a priori* information about the number of clusters one should expect in our dataset, we chose a hierarchical clustering approach for this analysis that is known to work especially well with smaller datasets.^25^ Agglomerative complete linkage clustering of Gower’s coefficient distances was performed using R package “cluster.” Two independent and well-established methods of verifying the quality of clustering solutions were used. Multi-scale bootstrap resampling was performed to assess the uncertainty of the clustering solution using R package “pvclust.”^26^ The approximately unbiased (AU) *P*-value was computed with 1000 times resampling and clusters with an AU *P*-value larger than .95 were set as significant modules. The optimal number of clusters (5) was determined by using the Silhouette coefficient method.^27^ The amount of time spent by a unique user reviewing a participant’s genetic results was defined as the time difference between the timestamp of any logged activity that immediately followed the last engagement with the *Genetics* section and the timestamp of the first logged access of this section. One-way ANOVA (Kruskal-Wallis test) was used to examine statistical significance of differences in time spent reviewing genetic test results between identified clusters of clinician engagement.

## RESULTS

### EHR access logs

In total, 34 access logs of clinician users were retrieved. They correspond to 30 unique clinician users who accessed the EHR’s *Genetics* section of 31 eMERGE participants (*n = *5 participants with positive results; *n = *26 participants with negative results). Of these 30 clinicians, only 13 went on to open at least one of the genetic test result documents (*n = *5 participants with positive results; *n = *8 participants with negative results), representing 1% of the 1071 study results that were uploaded onto iNYP.

### User characteristics and clinician workflows

User characteristics and workflow features are summarized in Table 1. Users were mostly (67%) males and had the following roles: physicians at the level of attending (*n = *14), fellow (*n = *4), and resident (*n = *9); nurse practitioners (*n = *3). Nineteen (63%) users practiced internal medicine (ie, “medicine”): nephrology (*n = *8); general internal medicine (*n = *5); gastroenterology (*n = *2); hematology/oncology (*n = *2); cardiology (*n = *1); rheumatology (*n = *1). The 11 remaining users were in specialties other than medicine: anesthesiology (*n = *4); surgery (*n = *3); radiology (*n = *2); neurology (*n = *1); pathology (*n = *1).

**Table 1.** User characteristics

Characteristics	Users *N = *30	**User session logs *N = *34** ^a^		
		User only accessed on the*Genetics* section *n = *20	User accessed the *Genetics* section and then clicked to view the genetic test results *n = *14	Total logs
Clinical role				
Attending-level physician (most senior)	14 (47%)	6	10	16 (47%)
Fellow-level physician	4 (13%)	3	2	5 (15%)
Resident-level physician	9 (30%)	9	1	10 (29%)
Nurse practitioner	3 (10%)	2	1	3 (<1%)
Clinical specialty				
Anesthesiology	4 (13%)	5	**–**	12 (35%)
Surgery	3 (9%)	3		
Radiology	2 (6%)	2		
Neurology	1 (3%)	1		
Pathology	1 (3%)	1		
Medicine	19 (63%)	8	14	22 (65%)
Time user accessed EHR				
During “regular” workhours^b^	23 (77%)	16	10	26 (77%)
During “on-call” hours^b^	6 (20%)	2	4	6 (18%)
During “regular” and “on-call” hours	1 (3%)	2	–	2 (6%)
Practice setting when user accessed EHR				
Outpatient/ambulatory	18 (60%)	10	9	19 (56%)
Inpatient/hospitalized	11 (37%)	9	3	12 (25%)
Both outpatient and inpatient settings	1 (3%)	1	2	3 (9%)
Context when user accessed EHR				
Around the time of a scheduled outpatient clinical encounter	11 (37%)	5	6	11 (32%)
Care team member of a hospitalized participants	11 (37%)	9	3	12 (35%)
Established outpatient provider	5 (17%)	3	5	8 (24%)
Not ascertained/unknown	3 (10%)	3	–	3 (9%)

a Corresponding to 31 study participants.

b “Regular” weekday hours (ie, Monday–Friday, 06:00–18:00); “on-call” (ie, evenings and weekends) hours.

Totals do not equal 100% due to rounding.

Seventy-seven percent of users (*n = *23/30) accessed the EHR during “regular” workhours (ie, Monday–Friday, 06:00–18:00) compared with 20% who accessed it during “on-call” (ie, evenings and weekends) hours, and one clinician who accessed the EHR of more than one participant during “regular” and “on-call” hours. Most (63%) clinicians accessed iNYP using hospital-supported platforms—the iNYP website or SCM Allscripts (*n = *5 and *n = *14, respectively), while 37% accessed iNYP through the university-supported EHR (Crown). Clinicians accessed the *Genetics* section in the following practice settings: outpatient (60%); inpatient/hospitalized (37%); both (3%).

Users accessed the *Genetics* section in the following contexts: around the date and time of a scheduled outpatient appointment with a participant (37%); as part of the inpatient care team during a participant’s hospitalization (37%); as a participant’s established outpatient provider (17%); not ascertained (10%).

Clinicians specialized in medicine (*n = *19) accessed the *Genetics* section of 20 eMERGE participants. Among them, 13 (68%) viewed at least one result document compared with 6 (32%) users who only accessed the *Genetics* section. Furthermore, all 5 positive results were viewed by a clinician specialized in medicine. In 2 cases, the clinician’s (ie, one nephrologist and a gastroenterologist) intervention was informed by the positive finding and documented as part of the participant’s outpatient encounter. The remaining 11 users, all from specialties other than medicine, accessed the *Genetics* section of 12 participants. None of these 11 users opened a genetic test result document, or accessed the *Genetics* section, for any of the participants with positive results.

### Engagement levels by user traits

Hierarchical clustering revealed 5 main types of clinician engagement with the genetic results, summarized in Table 2. Types A and B represent clinicians who in absolute majority of cases fully engaged with the genetic results and opened at least one of the documents. These types are represented primarily by attending physicians, specialized in medicine, (A) who were all part of the inpatient care team during a participant’s hospitalization and viewed genetic results mostly “on-call” and mostly using the hospital-supported EHR or (B) mostly viewed genetic results around the time of a participant’s scheduled outpatient appointment, during “regular” workhours, using the university-supported EHR. These clinicians, on average, spent 151 and 93 s reviewing genetic results, respectively. The remaining 3 cluster types (ie, C, D, and E) represent clinicians whose engagement with the genetic results was transient and limited to only accessing the *Genetics* section before transitioning to the next recorded activity, without viewing any documentation there. These types were represented by attending physicians of nonmedicine specialties who spent on average 17, 9, or 3 s accessing the *Genetics* section, respectively. Nonparametric ANOVA test of the time that clinicians spent accessing genetic results indicated that observed differences are significant (*P *< .001).

**Table 2.** Patterns of clinician engagement with EHR-integrated genetic test results

Engagement type (number of unique user session logs)	Engagement pattern	Entry viewed	Average time spent accessinggenetic testing results, s*
“Inpatient attending physicians” (A, *n *=* *5)	Mostly attending physicians; all of medicine specialties; all part of the inpatient care team during a participant’s hospitalization; mostly viewed genetic results during “on-call” hours^a^; mostly using the hospital-supported EHR^b^	*Mostly* accessed the *Genetics* section and then clicked to view the genetic test results	151
“Outpatient attending physicians” (B, *n* =* *11)	Mostly attending physicians; all of medicine specialties; mostly viewed genetic results around the date and time of a scheduled outpatient appointment with a participant; mostly during “regular” hours^a^; mostly using the university-supported EHR^b^	*Mostly* accessed the *Genetics* section and then clicked to view the genetic test results	93
“Non-medicine attending physicians” (C, *n* =* *3)	All attending physicians; mostly from surgery; accessing the *Genetics* section for unknown reasons or as an established outpatient provider of a participant; all during “regular” hours^a^; all using the hospital-supported iNYP website	*Only* accessed the *Genetics* section	17
“Outpatient trainees” (D, *n* =* *7)	Mostly residents; from diverse clinical specialties; mostly accessing the *Genetics* section around the date and time of a scheduled outpatient appointment with a participant; all during “regular” hours^a^; all using the hospital-supported EHR^b^	*Mostly* accessed the *Genetics* section	8
“Inpatient trainees” (E, *n* =* *8)	Mostly residents; from anesthesiology; all part of the inpatient care team during a participant’s hospitalization; mostly accessing the *Genetics* section during “regular” hours^a^; and mostly using the hospital-supported EHR^b^	*Only* accessed the *Genetics* section	3

“Regular” weekday workhours (ie, Monday–Friday, 06:00–18:00); “on-call” (ie, evenings and weekends) hours.

Hospital-supported EHR is *SCM Allscripts* and the university-supported EHR is *Crown*.

* *P* *< *.001.

## DISCUSSION

Participating eMERGE sites have devoted tremendous resources in development of infrastructures to support EHR integration and display of genomic data. In this study, we describe one institutional experience assessing clinicians’ engagement with unsolicited genetic test results displayed in the EHR as a proof of concept for using objective EHR user logs. We found that clinicians viewed only 1% of the total number of eMERGE results that were integrated in the EHR. The 13 users that went on to view the genetic test results were more likely to be senior clinicians (ie, attendings-level physicians) specialized in medicine (vs. anesthesiology, surgery, etc.). By categorizing each user by role, practice setting, and context for their involvement in a participant’s care, we also identified several patterns of user engagement with the EHR’s *Genetics* section and with unsolicited genetic test results based on user characteristics. The analysis also allowed us to assess the outcome of 2 users’ engagement with the genetic test results through the capture of 2 documented instances where the positive results influenced physician medical decision-making and subsequent management.

Similar to other EHR log studies, we were able to observe the clinician behaviors across a diverse set of clinical specialities.^5^^,^^6^^,^^8–14^^,^^28^^,^^29^ The log data provided us with enough identifiable information about the user to guide deeper chart review. This in turn allowed us to observe what users were doing when they accessed participants’ EHR data, giving us insights into their different workflows and tasks. Overall, few users in our study went on to view participants unsolicited genetic test results in the EHR, regardless of whether the findings were positive or negative. Unlike recent network studies on physicians’ attitudes toward unsolicited genetic test results, which use surveys and qualitative interviews,^30–32^ our study used objective and disease-agnostic data to assess the scope of clinician engagement with unsolicited genomic data. Furthermore, we make granular level observations of individual users in our study and compare which users viewed the study results versus who merely accessed the *Genetics* section before moving on to another section of the EHR. The insights into users’ specialties, levels of training, practice settings, and workflows, shed light onto users’ needs (ie, who needs to see what and when). For example, an attending physician, in internal medicine, seeing nonacute patients in an outpatient setting, may rely on a broad range of data for medical decision-making. In contrast, a junior resident-level physician in anesthesiology, performing pre-operative evaluations in acutely ill, hospitalized patients, may review EHR data in a more task-specific and narrow scope. These observations also give rise to questions that merit further investigation, such as the influence clinical role plays in the navigation and integration of patients’ EHR and genomic data, and the use of this information in users clinical decision-making.

The strengths of this study include the novel use of EHR access logs to assess clinicians’ engagement with unsolicited genomic data integrated in the EHR, at our institution. Our study provides valuable insights into the types of users who accessed the EHR’s *Genetics* section, but who may or may not have gone on to view the eMERGE genetic test results. Furthermore, this study is a proof of concept for the use of EHR log data as an objective assessment of the return on investment for integrating new types of clinical data into EHR platforms, and highlights the importance of addressing real-world barriers as part of EHR-integration efforts. An unexpected finding in our study was that despite iNYP’s reputed ease of use and clear clinical data displays, only a small portion of clinicians accessed the EHR-integrated eMERGE genetic test results. And, though it has been shown that certain types of EHR data goes unviewed by target users,^33^ our findings highlight an important, and still unresolved, question for genomic medicine—who are the clinicians that need to know about a patient’s actionable genomic findings? The scope of this problem is significant, and underscored by over a decade of work by national NHGRI-funded research networks, continuously focused on identifying sustainable ways to promote effective implementation of genomic findings into clinical care, through EHR-integration. Our findings underscore the urgency to identify these intended users as part of ongoing genomic implementation and EHR-integration research. Further study is needed into user characteristics, their role-specific informational and workflow needs, and the factors that influence their engagement with clinical and genomic data presented in the EHR. This information will inform the design of EHR data displays and clinical-decision support tools that are accessible, easy-to-consume, usable across diverse practice settings,^32^^,^^34^ and effective in promoting clinicians’ engagement and utilization of the genomic data.

## CONCLUSION

These data contribute important empirical knowledge on the application of objective EHR log data to evaluate clinician engagement with genomic data and with unsolicited findings displayed in the EHR, within a clinical context. It also provides insights into the informational and workflow needs of users and adds some valuable insights into types of activities that clinicians were engaged in when they accessed the unsolicited genetic test results. This study highlights opportunities to use EHR logs in ongoing EHR-integration efforts in both research and healthcare settings.

## FUNDING

The project was supported by grants from the National Institutes of Health through grants TL1TR001875 (JGN), 5U01HG008680-04 (eMERGE—Columbia University), and UL1TR001873 (CTSA) and the National Kidney Foundation’s Young Investigator Award (JGN).

## AUTHOR CONTRIBUTIONS

CW, SS, SB, GH, AG, WC, JW, DF, AF, and JGN conceived the study. CW, AF, and JGN designed the study. JGN, AF, and DF collected the data. JGN and AF analyzed the data. JGN, AF, and CW wrote the manuscript. All authors contributed to and discussed the results and critically reviewed the manuscript.

## DATA AVAILABILITY

The data underlying this article will be shared on reasonable request to the corresponding author.

## CONFLICT OF INTEREST STATEMENT

None declared.
